# Epidemiology and the Impact of Acute Kidney Injury on Outcomes in Patients with Rhabdomyolysis

**DOI:** 10.3390/jcm10091950

**Published:** 2021-05-01

**Authors:** Chien-Wen Yang, Si Li, Yishan Dong, Nitpriya Paliwal, Yichen Wang

**Affiliations:** 1 Renal Electrolyte and Hypertension Division, Hospital of the University of Pennsylvania, 3400 Spruce St, Philadelphia, PA 19104, USA; chien-wen.yang@pennmedicine.upenn.edu; 2 Department of Internal Medicine, Wright Center for Graduate Medical Education, 501 S Washington Ave, Scranton, PA 18505, USA; nitpriya26@gmail.com; 3 Department of Internal Medicine, Rochester General Hospital, 1425 Portland Ave, Rochester, NY 14621, USA; dongyishan89@gmail.com; 4 Mercy Internal Medicine Service, Trinity Health of New England, 271 Carew St, Springfield, MA 01104, USA; wangyichen25@gmail.com

**Keywords:** acute kidney injury, rhabdomyolysis, outcomes

## Abstract

Background: Currently, no large, nationwide studies have been conducted to analyze the demographic factors, underlying comorbidities, clinical outcomes, and health care utilization in rhabdomyolysis patients with and without acute kidney injury (AKI). Methods: We queried the National Inpatient Sample of Healthcare Cost and Utilization Project (HCUP) with patients with rhabdomyolysis from 2016 to 2018. The chi-squared test was used to compare categorical variables, and the adjusted Wald test was employed to compare quantitative variables. The logistic regression model was applied to calculate adjusted odds ratios (ORs) with 95% confidence intervals (95% CIs) to estimate the impact of AKI on outcomes in patients with rhabdomyolysis. Results: Among 111,085 rhabdomyolysis-related hospitalizations, a higher prevalence of AKI was noticed in older patients (mean age ± SD, 58.2 ± 21.6 vs. 53.8 ± 22.2), Medicare insurance (48.5% vs. 43.2%), and patients with a higher Charlson Comorbidity Index score (CCI 3–5, 15.1% vs. 5.5%). AKI was found to be independently associated with higher mortality (adjusted odds ratio [aOR] 3.33, 95% CI 2.33–4.75), longer hospital stays (adjusted difference 1.17 days, 95% CI: 1.00−1.34), and higher cost of hospital stay (adjusted difference $11,315.05, 95% CI: $9493.02–$13,137.07). Conclusions: AKI in patients hospitalized with rhabdomyolysis is related to adverse clinical outcomes and significant economic and survival burden.

## 1. Introduction

Rhabdomyolysis is a clinical condition characterized by the breakdown of skeletal muscle, which causes the release of intracellular enzymes to the bloodstream [1]. The first report of rhabdomyolysis is believed to be in the Old Testament. Jews, after consuming quail during the Exodus, were described to have developed symptoms similar to those of rhabdomyolysis [2]. Rhabdomyolysis-related hospitalizations were reported to be approximately 26,000 a year in the U.S. [3]. The etiologies of rhabdomyolysis are associated with direct or indirect muscle injuries, immobilization, seizure, infection, medication use, toxin exposure, underlying autoimmune diseases, and electrolyte imbalance. The clinical manifestation includes muscle ache, weakness, and dark urine. Serum creatinine kinase (CK) levels at the presentation of rhabdomyolysis are usually at least five times the upper limit of normal. The damaged muscle will also release myoglobin, causing typical urine findings [4,5].

AKI has been reported to be associated with 10–67% of patients with rhabdomyolysis, while 5–10% of the AKIs are caused by rhabdomyolysis [6,7,8]. Of patients with rhabdomyolysis, 4–8% were reported to require hemodialysis [8,9]. Myoglobin plays a crucial role in the underlying pathophysiology of the development of AKI. Myoglobin is released into the bloodstream from the intracellular space followed by membrane dysfunction and cell impairment. It is filtered freely and progressively concentrated in the urine after the solute, water-reabsorption process. The excessive amount of myoglobin obstructs the renal tubules and causes kidney injury. Besides myoglobin, the shifting of fluids from plasma to the injured muscle causes intravascular hypovolemia, leading to vasoconstriction by activating the sympathetic and renin-angiotensin system. The decrease in effective intravascular volume further increases the concentration of the myoglobin, which exacerbates the tubular obstruction. The release of intracellular materials can subsequently cause damage to the kidney by increasing free radicals and lipid peroxidation [10].

Several reports have shown that CK level is associated with the severity of kidney injury, while a recent retrospective study argues the correlation of this with renal replacement therapy (RRT) [11,12,13,14]. Scoring systems based on the risk factors were reported using age; gender; labs, including calcium, phosphate, creatinine, bicarbonate, potassium, lactate dehydrogenase, and CK level; and underlying etiologies to predict the risk of AKI with or without RRT [11,12]. Data from the National Inpatient Database suggest that the all-cause incidence of AKI has increased from 3.5% to 10.5%, with secondary diagnosis of AKI increasing 188.8% and primary diagnosis 66.1% from 2005 to 2014 [15]. However, there are no large clinical data to analyze the effect of AKI on rhabdomyolysis, and much uncertainty still exists about whether AKI is an independent factor of adverse outcomes and healthcare resource utilization in patients hospitalized with rhabdomyolysis.

## 2. Materials and Methods

### 2.1. Data Source

We extracted our study cohort from the Nationwide Inpatient Sample (NIS) and the National Inpatient Sample of Healthcare Cost and Utilization Project (HCUP) compiled by the Agency for Healthcare Research and Quality [16]. The NIS, the largest inpatient care database in the United States, stores data from 5 to 8 million hospital stays in about 1000 hospitals across the country each year. Its participating hospital pool approximates a 20% stratified sample of U.S. community hospitals, resulting in a sampling frame comprising about 90% of all hospital discharges in the U.S. This allows for the calculation of precise, weighted, nationwide estimates. The NIS also stores information on clinical and resource utilization information that is included in a discharge abstract. It contains clinical and nonclinical data elements for each hospitalization, including patient demographic characteristics, expected payment source, length of stay, severity, comorbidity measures, etc. All data elements that directly identify an individual have been removed for confidentiality purposes and do not require institutional review board (IRB) approval or exempt determination. We have signed the HCUP Data Use Agreement which strictly prohibits us from making any effort to determine the identity of any person contained in the data, including patients, physicians and other providers.

### 2.2. Study Population

From 2016 to 2018, we queried the NIS database for hospitalizations with primary diagnosis codes for rhabdomyolysis International Classification of Diseases, Tenth Revision-Clinical Modification (ICD−10-CM) code M62.82 and secondary codes for AKI (N17). We excluded patients under the age of 18 years. We then extracted baseline characteristics of the study population. Patient-level characteristics included age, sex, race, quartile classification of median household income according to postal (ZIP) code, and primary payer (Medicare/Medicaid, private insurance, self-pay, no charge). Hospital-level characteristics included hospital location (urban, rural), hospital bed size (small, medium, large), region (Northeast, Midwest/North Central, South, West), and teaching status. We defined illness severity and the likelihood of death using the CCI, which draws on principal and secondary ICD−10-CM diagnosis codes, the details of diagnosis codes are included in the Tables S1 and S2.

### 2.3. Statistical Analysis

Statistical analysis was performed using Stata 16.0 (StataCorp LLC, College Station, TX, USA). Data were treated as survey data and weighted using the discharge-level weight variable (DISCWT) and the built-in survey data designation to create national estimates. All statistics presented here represent weighted estimates unless explicitly stated. Categorical variables were compared using a chi-square test, and comparison of two means was performed using an adjusted Wald test. Multivariate regression models were built to calculate adjusted odds ratio (OR) with 95% confidence intervals (95% CIs) to estimate the impact of AKI on rhabdomyolysis using the following method: Univariate regression analyses on possible confounding factors were used to calculate the unadjusted odds ratio. Those with *p*-values less or equal to 0.2 were chosen as potential confounding factors. Variables considered clinically relevant to the outcomes were chosen as potential confounding factors regardless of *p*-values on the previous step. Potential confounding factors were then included in multivariate logistic regressions to analyze relationships between AKI and outcomes. Factors with *p* < 0.05 in the multivariable model were considered significant. The Variance Inflation Factor tested collinearity in the multivariable models and none of the predictors had a variance inflation factor greater than five, indicating that there is no collinearity of the predictors. A cutoff of *p* < 0.05 was used for statistical significance.

### 2.4. Aim of the Study

Our clinical interests were to: (1) evaluate the occurrence of AKI in rhabdomyolysis-related hospitalization; (2) compare the demographic factors and underlying comorbidities in rhabdomyolysis patients with and without AKI; and (3) assess the effect of AKI on rhabdomyolysis hospitalizations, including the in-hospital mortality, length of stay, hospital cost and charge, and development of complications.

## 3. Results

### 3.1. Baseline Demographics and Patient Characteristics

Of 107,001,355 hospitalizations from 2016 to 2018 in the U.S., 90,879,561 were adults. An estimated 111,085 admissions had a principal discharge diagnosis of rhabdomyolysis, 26,650 (24.0%) of whom had a secondary diagnosis of AKI. Table 1 summarizes the baseline characteristics of demographics, socioeconomics, and comorbidities between patients with and without AKI.

Compared to patients without AKI, patients with AKI were more likely to be older (mean age ± SD, 58.2 ± 21.6 vs. 53.8 ± 22.2, *p* < 0.001), more likely to be older than 65 (43.5% vs. 37.3%, *p* < 0.001), and less likely to be female (29.2% vs. 38.1%, *p* < 0.001). Patients with AKI, compared to those without, were more likely from large hospitals (45.4% vs. 42.4%, *p* < 0.001) and from the Midwest region (20.9% vs. 18.9%, *p* < 0.001). Regarding socioeconomic characteristics, patients with AKI were more likely to have Medicare (48.5% vs. 43.2%, *p* < 0.001) and to belong to the first quartile of median household income (35.5% vs. 33.5%, *p* < 0.001). Regarding disposition, patients with AKI were more likely to be transferred to a short-term hospital or facility and more likely to be discharged with home health care.

Patients with AKI compared to patients without AKI were more likely to have a higher CCI score (CCI 3–5, 15.1% vs. 5.5%, *p* < 0.001) and more chronic comorbidities, including chronic kidney disease, congestive heart failure, coronary artery disease, diabetes mellitus, atrial fibrillation, and hyperlipidemia. Patients with AKI also had a higher rate of opioid use, cocaine use, and a traumatic event being recorded as a diagnosis. The other characteristics were similar between the two patient groups or had differences numerically too small to be clinically significant.

### 3.2. Impact of AKI on Outcomes

**Figure 1:**
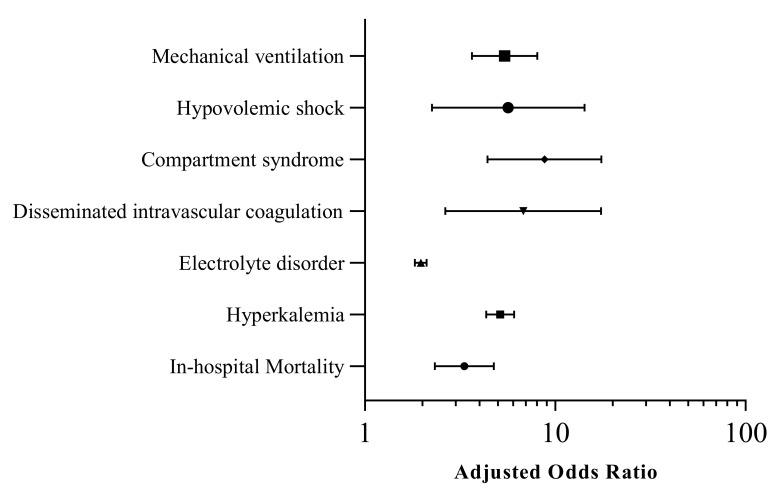
Multivariable logistic regression model showing effect of AKI on mortality and complications in rhabdomyolysis-related hospitalizations.

We then analyzed the impact of AKI on clinical outcomes. (Table 2) After adjusting for hospital and individual level of confounders, AKI was found to be independently associated with a higher in-hospital mortality (adjusted odds ratio [aOR] 3.33, 95% CI 2.33−4.75, *p* < 0.001) and complications, including hyperkalemia (aOR 5.14, 95% CI 4.34−6.09, *p* < 0.001), electrolyte disorders (aOR 1.97, 95% CI 1.83–2.11, *p* < 0.001), disseminated intravascular coagulation, compartment syndrome, hypovolemic shock, and mechanical ventilation. (Figure 1). We also found 1.9% of patients who developed AKI required hemodialysis during the rhabdomyolysis related hospitalization. After adjusting for patient and hospital level of potential confounders, younger age (aOR 0.96, 95% CI 0.95–0.98), chronic kidney disease (aOR 5.32, 95% CI 2.75–10.26) and hypertension (aOR 1.84, 95% CI 1.07–3.16) were found to be associated with a higher risk of AKI requiring hemodialysis.

Three markers—length of stay (LOS), total hospitalization charges, and total hospitalization costs have been used to estimate the effect of AKI on resource utilization. The overall mean LOS was 4.3 ± 4.6 days (interquartile range [IQR]: 2–5) in patients hospitalized for rhabdomyolysis, 4.0 ± 4.0 days (IQR: 2–5) for patients without AKI, and 5.4 ± 6.0 days (IQR: 2–6) for patients with AKI. After adjusting for confounders, patients with AKI had a longer adjusted mean LOS compared to patients without AKI (adjusted difference 1.17 days, 95% CI: 1.00–1.34, *p* < 0.001). (Table 3).

Mean total hospitalization charges were found to be $31,489.3 ± $40,672.9 (IQR: $13,209.0–$36,942.0) for the overall study population, $28,399.6 ± $29,994.7 (IQR: $12,779.0–$34,273.0) for patients without AKI, and $41,296.0 ± $62,633.5 (IQR: $14,752.0–$46,142.0) for patients with AKI. After adjusting for the confounders, total hospitalization charges were significantly higher for patients with AKI compared to patients without AKI (adjusted difference $11,315.05, 95% CI: $9493.02–$13,137.07, *p* < 0.001). Similar results were found when examining total hospitalization costs, with the overall study population, patients without AKI and patients with AKI having mean total hospitalization costs of $7659.6 ± $8655.9 (IQR: $3687.0–$8925.0), $6883.5 ± $6454.8 (IQR: $3547.0–$8253.0), and $10,122.8 ± $13,134.6 (IQR: $4272.0-$11,389.0), respectively. Patients with AKI had significantly higher mean total hospitalization costs compared to patients without AKI (adjusted difference $2785.00, 95% CI: $2394.05–$3175.95, *p* < 0.001).

## 4. Discussion

Analysis of more than 100,000 national rhabdomyolysis-associated hospitalizations has shown that AKI is associated with a more severe hospital course (as measured by the development of complications), increased in-hospital mortality, and more frequent health-care resource utilization. These differences remain the same after adjusting for other variables.

The overall incidence of AKI in rhabdomyolysis-associated hospitalizations in our study is 24%. A wide variety of incidence (10–67%) was reported from previous observations, which were based on small population studies and may vary according to the etiology of rhabdomyolysis in the patient database being studied [6,7,8]. Candela et al. conducted a multicenter retrospective study of 387 patients with severe rhabdomyolysis (CK > 5000 U/L) that demonstrated an incidence of AKI of 81.4% [17]. The differences may reflect the heterogeneity of the population studied in previous studies and various diagnostic criteria; however, this study is the first to our knowledge to address the impact of AKI on rhabdomyolysis at the national level.

We concluded that older patients with rhabdomyolysis have a higher tendency to have AKI. McMahon et al. reviewed 2371 patients with rhabdomyolysis and concluded age as a significant factor associated with AKI requiring RRT. The mean age of that study’s population was 50.7 years, which is close to the 54.8 mean age in the present study [12] Rodriguez et al. retrospectively analyzed 126 patients with severe rhabdomyolysis, which showed age to not be significantly related to AKI. The mean age of that study’s population was 54 years [18]. The explanation of this different finding may be the smaller sample size and that the study focused on only patients with severe rhabdomyolysis. Based on the results of the present study, we identify elder age as a potential significant factor associated with AKI in rhabdomyolysis patients. In a retrospective chart review study of geriatric population, fall with or without immobilization was found to be the most frequent cause of rhabdomyolysis (56.9%) [19]. The frailty status among rhabdomyolysis patients has not been well studied despite its importance for prognostication. with a large subgroup analysis with stratification of the severity of rhabdomyolysis and frailty index score being warranted in future studies.

The female gender is less likely to be associated with AKI in patients with rhabdomyolysis according to the present study. This result is different from the result McMahon et al. reported, which were that female gender is associated with a higher risk of AKI requiring RRT [12]. McMahon enrolled 622 female patients, while the present study analyzed 39,955 patients. Thus, the role of gender in patients with rhabdomyolysis warrants future investigation.

We found AKI to be associated with a higher rate of opioid and cocaine use in patients with rhabdomyolysis. Opioid overdose can cause hypotension, leading to rhabdomyolysis and AKI [20] Opioid withdrawal was previously reported to be associated with seizure, but the correlation with AKI is unclear [21]. Roth et al. identified 39 patients with acute rhabdomyolysis after cocaine use. Thirteen (33%) of them had AKI. Cocaine can stimulate a sympathetic system response by catecholamine uptake inhibition and release of norepinephrine and epinephrine, and cause vasoconstriction at the renal level [10,22,23]. Importantly, in recent years, the emerging use of levamisole-adulterated cocaine has been found to be associated with ANCA-associated vasculitis and immune complex glomerulonephritis [24]. Other than direct nephrotoxicity, findings including vasculitis, vascular infarction, and thrombosis were revealed in kidney biopsies reported by Pendergraft et al. [25]. This direct nephrotoxic effect may be independent from myoglobinuria-induced tubular necrosis in patients with rhabdomyolysis. Our study broadly supports evidence from previous findings by Lau-Hing Yim et al. [26]. The author found illicit drug use was independently associated with higher severity of rhabdomyolysis and a greater likelihood of AKI and renal replacement therapy requirements. Together these results serve as a stark reminder of the need to investigate this public health concern. It also provides important insights into future clinical practice, that positive clinical history or screening of substance use should raise a suspicion of AKI in patients with rhabdomyolysis.

Patients with a higher CCI score have a higher tendency to have AKI. Based on the present study, atrial fibrillation, coronary artery disease, congestive heart failure, disseminated intravascular coagulation, compartment syndrome, and hypovolemic shock are shown to be associated with AKI in patients with rhabdomyolysis. The possible underlying mechanisms include hemodynamic instability resulting in AKI and decreased renal perfusion leading to the accumulation of myoglobin, which damages tubular cells [10,27]. Diabetes and chronic kidney disease are also found to be related to AKI in patients with rhabdomyolysis. This could be explained by losing the tubule-glomerular feedback, which leads to hypoperfusion in the setting of rhabdomyolysis. Statin-induced rhabdomyolysis may be the reason hyperlipidemia contributes to AKI, but the medication data are unavailable in the current database [10,28]. Research has shown that low socioeconomic status is related to multimorbidity. This could explain why patients with rhabdomyolysis in the first quartile of median household income tend to have more AKI [29].

Among the discharged patients, there are significant economic and survival burdens associated with rhabdomyolysis with AKI. Our study supports evidence from previous observations. AKI has been reported to be associated with higher mortality in patients with all-cause and combat-injury-induced rhabdomyolysis [30,31]. Another important finding was that AKI is independently associated with the increased requirement of mechanical ventilation. A strong relationship between prolonged ventilator dependency and development of AKI in patients with rhabdomyolysis has also been reported by Candela et al. [17]. Prolonged hospital stay is known to increase the risk of exposure to nosocomial infection and thromboembolism. Ventilator dependency is also an identified risk factor of ventilator-associated pneumonia, which is a large disease burden and results in morbidity and mortality [32,33,34]. It will also be valuable to explore the differences of impact on clinical and economic outcomes between rhabdomyolysis associated AKI and AKI related to other causes. Importantly, serial laboratory data and the use of medications, particularly some antihypertensive agents, diuretics, drugs mainly excreted by the kidney, should be taken in context to evaluate the differences. We hope that the need and motivation for further randomized controlled prospective trials and observational studies are emphasized through our investigation.

The strength of our study comes from the fact of using a national-level database covering patients in both academic and private institutions, that it is designed to be representative of healthcare use overall, and that its size and scope make it ideal for deriving national estimates and descriptions. The ICD−10 codes we used were based on previous validation studies, to identify acute kidney injury and other comorbidities [35,36]. The presence or absence of ICD−10 code N17x was found to have specificity greater than 95% in hospital admissions. However, certain limitations do exist in the current study, given the nature of administrative data:

(1) Some clinical components (e.g., vital signs and laboratory values) are not captured. Thus, we cannot classify the patients based on the severity of CK level or the stage of AKI. Also, CK level was not routinely checked in all patients seeking treatment at the hospital, therefore, a number of patients may have developed rhabdomyolysis without being diagnosed.
(2) The extracted data are based on diagnostic codes from each hospitalization. Thus, we are unable to determine the causal inference of the electrolyte imbalance and rhabdomyolysis.
(3) The NIS and HCUP database did not recruit patient-level data. Thus, we are unable to exclude patients with multiple admissions.
(4) The database represents only hospitalized patients, including patients with mild rhabdomyolysis treated in the emergency room or outpatient setting.
(5) Even after adjusting for the potential confounders, it is impossible to establish causality due to the possibility of residual confounding. Further randomized trials are needed to allow definitive conclusions concerning the relationships between AKI and outcomes.

## 5. Conclusions

Based on the large, nationwide database analysis, we concluded that AKI is an independent predictor of higher mortality and more complications among patients admitted to the hospital with rhabdomyolysis. Besides, AKI is associated with a significant economic and survival burden, including LOS and total costs and charges. Prospective studies would be useful to determine the effectiveness of early initiation of therapeutic approaches on kidney function preservation to reduce comorbidities and improve outcomes.

## Figures and Tables

**Table 1:** Baseline characteristics of rhabdomyolysis-related hospitalizations with and without AKI.

Characteristics	All Rhabdomyolysis (*n* =) (100%)	With Acute Kidney Injury (*n* =) (%)	Without Acute Kidney Injury (*n* =) (%)	*p*-Value
Age, y, mean ± SD	54.8 ± 22.9	58.2 ± 21.6	53.8 ± 22.2	<0.001
Age, [*n* (%)]				<0.001
18−44	43,160 (38.9)	8280 (31.1)	34,880 (41.3)	
45−64	24,815 (22.3)	6775 (25.4)	18,040 (21.4)	
≥65	43,110 (38.8)	11,595 (43.5)	31,515 (37.3)	
Female gender [*n* (%)]	39,955 (36.0)	7775 (29.2)	32,180 (38.1)	<0.001
Race [*n* (%)]				0.1121
White	64,470 (59.7)	15,290 (59.4)	49,180 (59.8)	
Black	26,280 (24.3)	6600 (25.6)	19,680 (23.9)	
Hispanic	11,550 (10.7)	2620 (10.2)	8930 (10.9)	
Asian or Pacific Islander	1985 (1.8)	425 (1.7)	1560 (1.9)	
Native American	490 (0.5)	100 (0.4)	390 (0.5)	
Other	3225 (3.0)	725 (2.8)	2500 (3.0)	
Primary payer [*n* (%)]				<0.001
Medicare	49,350 (44.5)	12,920 (48.5)	36,430 (43.2)	
Medicaid	20,965 (18.9)	5320 (20.0)	15,645 (18.6)	
Private	26,035 (23.5)	4705 (17.7)	21,330 (25.3)	
Self-pay	9390 (8.5)	2495 (9.4)	6895 (8.2)	
Other	5200 (4.7)	1180 (4.4)	4020 (4.8)	
Median household income in the patient’s zip code [*n* (%)]				<0.001
First quartile	36,610 (34.0)	9150 (35.5)	27,460 (33.5)	
Second quartile	27,595 (25.6)	6730 (26.1)	20,865 (25.5)	
Third quartile	23,685 (22.0)	5655 (21.9)	18,030 (22.0)	
Fourth quartile	19,855 (18.4)	4250 (16.5)	15,605 (19.0)	
Hospital bed size [*n* (%)]				<0.001
Small	27,575 (24.8)	6240 (23.4)	21,335 (25.3)	
Medium	35,615 (32.1)	8310 (31.2)	27,305 (32.3)	
Large	47,895 (43.1)	12,100 (45.4)	35,795 (42.4)	
Hospital region [*n* (%)]				<0.001
Northeast	23,180 (20.9)	4940 (18.5)	18,240 (21.6)	
Midwest	21,535 (19.4)	5575 (20.9)	15,960 (18.9)	
South	46,765 (42.1)	11,320 (42.5)	35,445 (42.0)	
West	19,605 (17.7)	4815 (18.1)	14,790 (17.5)	
Teaching Hospital [*n* (%)]	69,275 (62.4)	16,675 (62.6)	52,600 (62.3)	**0.7321**
Hospital urban location [*n* (%)]	97,825 (88.1)	23,425 (88.0)	74,400 (88.1)	**0.6737**
Charlson Comorbidity Index score [*n* (%)]				<0.001
0–2	101,005 (90.9)	22,075 (82.8)	78,930 (93.5)	
3–5	8685 (7.8)	4025 (15.1)	4660 (5.5)	
≥6	1395 (1.3)	550 (2.1)	845 (1.0)	
Length of stay, d, mean ± SD	4.3 ± 4.6	5.4 ± 6.0	4.0 ± 4.0	<0.001
Length of stay group [*n* (%)]				<0.001
0–3	60,335 (54.3)	12,100 (45.4)	48,235 (57.1)	
4–7	38,595 (34.7)	9700 (36.4)	28,895 (34.2)	
≥8	12,155 (10.9)	4850 (18.2)	7305 (8.7)	
Charge of hospitalization in U.S. $, mean ± SD	31,489.3 ± 40672.9	41,296.0 ± 62,633.5	28,399.6 ± 29,994.7	<0.001
Cost of hospitalization in U.S. $, mean ± SD	7659.6 ± 8655.9	10,122.8 ± 13,134.6	6883.5 ± 6454.8	<0.001
Disposition [*n* (%)]				<0.001
Routine discharge	58,270 (52.5)	12,835 (48.2)	45,435 (53.9)	
Transfer to Short-term Hospital	2095 (1.9)	665 (2.5)	1430 (1.7)	
Transfer to Facility	35,100 (31.6)	8960 (33.6)	26,140 (31.0)	
Discharge with Home Health Care (HHC)	9555 (8.6)	2580 (9.7)	6975 (8.3)	
Against Medical Advice (AMA)	5320 (4.8)	1235 (4.6)	4085 (4.8)	
Died	675 (0.6)	360 (1.4)	315 (0.4)	
Chronic kidney disease [*n* (%)]	11,470 (10.3)	6400 (24.0)	5070 (6.0)	<0.001
Hypertension [*n* (%)]	40,945 (36.9)	9130 (34.3)	31,815 (37.7)	<0.001
Congestive heart failure [*n* (%)]	8415 (7.6)	3030 (11.4)	5385 (6.4)	<0.001
Coronary artery disease [*n* (%)]	13,645 (12.3)	4175 (15.7)	9470 (11.2)	<0.001
Diabetes mellitus [*n* (%)]	20,120 (18.1)	6505 (24.4)	13,615 (16.1)	<0.001
Atrial fibrillation [*n* (%)]	9645 (8.7)	3060 (11.5)	6585 (7.8)	<0.001
Hyperlipidemia [*n* (%)]	27,210 (24.5)	7685 (28.8)	19,525 (23.1)	<0.001
Dementia [*n* (%)]	8415 (7.6)	1920 (7.2)	6495 (7.7)	0.2347
HIV infection [*n* (%)]	455 (0.4)	150 (0.6)	305 (0.4)	0.0476
Alcohol use [*n* (%)]	12,885 (11.6)	3090 (11.6)	9795 (11.6)	0.9908
Opioid use [*n* (%)]	4835 (4.4)	1685 (6.3)	3150 (3.7)	<0.001
Cannabis use [*n* (%)]	7940 (7.2)	2060 (7.7)	5880 (7.0)	0.0634
Cocaine use [*n* (%)]	6140 (5.5)	2000 (7.5)	4140 (4.9)	<0.001
Trauma [*n* (%)]	15,975 (14.4)	4125 (15.5)	11,850 (14.0)	0.0099

**Table 2:** Univariate and multivariable logistic regression model showing effect of AKI on mortality and complications in rhabdomyolysis-related hospitalizations.

Variables	Crude Odds Ratio (95% Confidence Interval)	*p*-Value	Adjusted Odds Ratio (95% Confidence Interval)	*p*-Value
In-hospital Mortality	3.66 (2.61–5.13)	<0.001	3.33 (2.33–4.75)	<0.001
Hyperkalemia	6.12 (5.24–7.15)	<0.001	5.14 (4.34–6.09)	<0.001
Electrolyte disorder	1.94 (1.82–2.07)	<0.001	1.97 (1.83–2.11)	<0.001
Disseminated intravascular coagulation	6.88 (2.61–18.12)	<0.001	6.79 (2.65–17.39)	<0.001
Compartment syndrome	6.85 (3.52–13.34)	<0.001	8.78 (4.41–17.47)	<0.001
Hypovolemic shock	6.35 (2.56–15.74)	<0.001	5.66 (2.25–14.25)	<0.001
Mechanical ventilation	5.78 (3.94–8.49)	<0.001	5.42 (3.65–8.05)	<0.001

**Table 3:** Univariate and multivariable logistic regression model showing effect of AKI on resource utilization in rhabdomyolysis-related hospitalizations.

Variables	Crude Coefficient (95% Confidence Interval)	*p*-Value	Adjusted Coefficient (95% Confidence Interval)	*p*-Value
Additional length of hospital stays (d)	1.40 (1.23–1.57)	<0.001	1.17 (1.00–1.34)	<0.001
Additional total hospitalization charges	12,896.32 (11,134.27–14,658.37)	<0.001	11,315.05(9493.02–13,137.07)	<0.001
Additional total hospitalization costs	3239.33 (2862.80–3615.87)	<0.001	2785.00 (2394.05–3175.95)	<0.001

## Data Availability

Publicly available datasets were analyzed in this study. This data can be found here: https://www.hcup-us.ahrq.gov/db/nation/nis/nisdbdocumentation.jsp. The site was accessed on 27 January 2021.

## Supplementary Materials

The following are available online at https://www.mdpi.com/article/10.3390/jcm10091950/s1, Table S1. International Classification of Diseases (ICD−10) code for medical comorbidities; Table S2. International Classification of Diseases (ICD−10) code for complications
