# Supplementary material for: Epidemiology and the Impact of Acute Kidney Injury on Outcomes in Patients with Rhabdomyolysis
Source: J Clin Med. 2021 May 1;10(9):1950. doi: 10.3390/jcm10091950 (PMC8125267; doi:10.3390/jcm10091950)
Supplement: Supplementary file 1 [file jcm-10-01950-s001.zip › jcm-1162873-supplementary.pdf]

Supplement Table 1. International Classification of Diseases (ICD-10) code for medical comorbidities

Hypertension	I10 Essential (primary) hypertension
CKD	N18 Chronic kidney disease (CKD)<br>N18.1 Chronic kidney disease, stage 1<br>N18.2 Chronic kidney disease, stage 2 (mild)<br>N18.3 Chronic kidney disease, stage 3 (moderate)<br>N18.30 Chronic kidney disease, stage 3 unspecified<br>N18.31 Chronic kidney disease, stage 3a<br>N18.32 Chronic kidney disease, stage 3b<br>N18.4 Chronic kidney disease, stage 4 (severe)<br>N18.5 Chronic kidney disease, stage 5<br>N18.6 End stage renal disease<br>N18.9 Chronic kidney disease, unspecified
Congestive heart failure	I50 Heart failure<br>I09.81 Rheumatic heart failure<br>I11.0 Hypertensive heart disease with heart failure<br>I13.0 Hypertensive heart and chronic kidney disease with heart failure and stage 1 through stage 4 chronic kidney disease, or unspecified chronic kidney disease<br>I13.2 Hypertensive heart and chronic kidney disease with heart failure and with stage 5 chronic kidney disease, or end stage renal disease
Coronary artery disease	I25 Chronic ischemic heart disease
Diabetes mellitus	E08 Diabetes mellitus due to underlying condition<br>E09 Drug or chemical induced diabetes mellitus<br>E10 Type 1 diabetes mellitus<br>E11 Type 2 diabetes mellitus<br>E13 Other specified diabetes mellitus
Atrial fibrillation	I48 Atrial fibrillation and flutter
Hyperlipidemia	E78 Disorders of lipoprotein metabolism and other lipidemia
Dementia	F03.9 Unspecified dementia
HIV	B20 Human immunodeficiency virus [HIV] disease
Alcohol use	F10 Alcohol related disorders
Opioid use	F11 Opioid related disorders
Cannabis use	F12 Cannabis related disorders
Cocaine use	F14 Cocaine related disorders
Trauma	S00-S09 Injuries to the head<br>S10-S19 Injuries to the neck<br>S20-S29 Injuries to the thorax<br>S30-S39 Injuries to the abdomen, lower back, lumbar spine, pelvis and external genitals<br>S40-S49 Injuries to the shoulder and upper arm

	S50-S59 Injuries to the elbow and forearm<br>S60-S69 Injuries to the wrist, hand and fingers<br>S70-S79 Injuries to the hip and thigh<br>S80-S89 Injuries to the knee and lower leg<br>S90-S99 Injuries to the ankle and foot<br>T07-T07 Injuries involving multiple body regions<br>T14-T14 Injury of unspecified body region

Supplement Table 2. International Classification of Diseases (ICD-10) code for complications

Hyperkalemia	E875 Hyperkalemia
Electrolyte abnormality	E870 Hyperosmolality and hypernatremia<br>E871 Hypo-osmolality and hyponatremia<br>E875 Hyperkalemia<br>E876 Hypokalemia<br>E833 Disorders of phosphorus metabolism and phosphatases<br>E834 Disorders of magnesium metabolism<br>E835 Disorders of calcium metabolism
DIC	D65 Disseminated intravascular coagulation [defibrination syndrome]
Hypovolemic shock	R571 Hypovolemic shock
Mechanical ventilation	0BH17EZ Insertion of Endotracheal Airway into Trachea, Via Natural or Artificial Opening<br>5A1935Z Respiratory Ventilation, Less than 24 Consecutive Hours<br>5A1945Z Respiratory Ventilation, 24-96 Consecutive Hours<br>5A1955Z Respiratory Ventilation, Greater than 96 Consecutive Hours
Hemodialysis	Z99.2 Dependence on renal dialysis<br>Z49.01 Encounter for fitting and adjustment of extracorporeal dialysis catheter<br>5A1D70Z Performance of Urinary Filtration, Intermittent, Less than 6 Hours Per Day<br>5A1D80Z Performance of Urinary Filtration, Prolonged Intermittent, 6-18 hours Per Day<br>5A1D90Z Performance of Urinary Filtration, Continuous, Greater than 18 hours Per Day
